# NCCN Harmonized Guidelines for Sub-Saharan Africa: A Collaborative Methodology for Translating Resource-Adapted Guidelines Into Actionable In-Country Cancer Control Plans

**DOI:** 10.1200/GO.20.00436

**Published:** 2020-09-24

**Authors:** Benjamin O. Anderson

**Affiliations:** ^1^ Breast Health Global Initiative, Fred Hutchinson Cancer Research Center, University of Washington, Seattle, WA

In high-income countries (HICs) and some upper-middle-income countries, deaths from cancer are now more common than those from cardiovascular disease; this transformation relates to a demographic transition to cancer becoming the predominant cause of mortality among people ages 35-70 years.^1^ As cardiovascular disease continue to decrease in HICs and upper-middle-income countries, cancer will likely become the leading cause of death in these regions. In parallel with these changes, cancer has been an increasing issue in sub-Saharan Africa (SSA), where infectious diseases and maternal mortality are better controlled but, at the same time, patients with cancer have accentuated mortality rates relating to disorganized or inaccessible cancer diagnoses and treatment services. As a result, global cancer control efforts cannot rely purely on risk factor reduction and will require systematic improvements in health care access and quality.^2^ For these reasons, SSA countries are wise to seek counsel on how best to build capacity to receive and care for the rising tide of patients with cancer, applying economically realistic and sustainable approaches as part of a broader strategy for overall health systems strengthening. These conversations are complex and require thoughtful dialogue with ministries of both health and finance.

To estimate the cost of cancer care infrastructure, personnel, medicines, and other consumable resources, disease-based cancer management guidelines are required as a framework for organizational planning. High-quality, evidence-based guidelines have been developed in HICs on the basis of strong scientific evidence from randomized trials and other clinical studies performed in these same HICs, where resource constraints are not considered a primary limitation for health care delivery. Because most HIC guidelines, including the original parent guidelines of the National Comprehensive Cancer Network (NCCN), previously provided no prioritization schemes for strategic implementation, they had limited utility in counties with under-resourced health care services, where the care must be both therapeutically efficacious and cost effective for the delivery approaches to be valuable and sustainable over time. It is a false dichotomy to suggest that, if countries cannot afford to set up all health care infrastructure in the way that HICs do, they therefore cannot manage cancer. Health systems strengthening is not an all-or-none proposition.

The concept of resource-stratified guidelines to provide an organized methodology for prioritizing resource acquisition for cancer treatment was established by the Breast Health Global Initiative (BHGI) through a series of Global Summits beginning in 2002.^3^ The resource-stratified guideline approach was later adopted by NCCN, ASCO, the World Bank in their publication “Cancer: Disease Control Priorities, Third Edition,”^4,5^ and—ultimately—by WHO through the 2017 Cancer Prevention and Control Resolution (Resolution 70.12) adopted at the 70th World Health Assembly.^6^

There is a common misconception that resource-stratified guidelines effectively lower the bar for cancer care delivery in settings where treatment is already inadequate. To the contrary, resource-stratification strengthens health care systems by identifying those essential resources and services that act as a foundation for building organized delivery systems that are functional and sustainable. When a government purchases or acquires equipment for which the necessary infrastructure is lacking, critical funds are wasted, as the desired services never become fully functional. Strategic planning guided by resource-adapted implementation principles is a core value for durable health care deployment.

NCCN’s initial foray into resource-stratified guidelines was the development of NCCN Frameworks for Resource Stratification (hereafter, NCCN Frameworks) that stratify selected HIC-appropriate NCCN Clinical Practice Guidelines in Oncology (parent NCCN guidelines) into four resource environments using a methodology based on the approach previously developed by BHGI.^7^ Specifically, NCCN identified specific NCCN guidelines appropriate for stratification, then called on a subgroup of multidisciplinary experts chosen from the NCCN guideline panels to implement the stratification progress. NCCN funds all guideline development activities from NCCN member institution dues with no contributions from industry and follows standardized conflict of interest policies; these policies mean that all panelists are required to update a conflict of interest report at least every 6 months with strict criteria that, if not met, will prohibit that individual’s participation in guideline-related activities. Each panelist was educated about the principles and practice of resource stratification and was then asked to assign a priority to each guideline recommendation according to resource availability. These assignments were used to draft an initial resource-stratified framework to be reviewed by the full guidelines panel for appropriateness, comment, and possible revision. After the panel agreed on the resource-stratified framework for each selected guideline, a preliminary version of the NCCN Framework was developed and circulated to external expert reviewers with disease-specific experience at various resource levels. The additional comments from these reviewers were evaluated and additional revisions were made before each resource-stratified NCCN Framework was finalized on the NCCN website. As of August 2020, 16 finalized and three preliminary NCCN Frameworks have been posted on the NCCN website.^8^

Resource-stratified guidelines provide a general framework for stepwise implementation at any resource level and are neither prescriptive nor rigid in defining next steps for a specific health care system or environment. As such, resource-stratified guidelines can be challenging to use in specific situations, because they are more conceptual than applied. A 2014 literature review showed that BHGI guidelines were increasingly referenced in the global health literature, but it also found that few articles (< 6%) referenced the guidelines as a framework for implementation of a specific cancer plan.^9^ To move from theoretical frameworks based on principles of resource-stratification to practical real-world applications, new tools and approaches are required.

A novel, regionally specific approach for adapting resource-stratified guidelines to real-world application is described in the accompanying article by Mutebi et al^10^ in *JCO Global Oncology*, which specifically addresses SSA issues and needs. The African Cancer Coalition (ACC) was established in 2016 under the leadership of the former Health Minister of Nigeria, Isaac Adewole, and the director of the Uganda Cancer Institute, Jackson Orem, as a regional collaboration to develop comprehensive standard cancer treatment guidelines that are tailored for SSA use. Bringing together cancer experts from 12 SSA countries, the ACC worked in collaboration with NCCN and the American Cancer Society through a series of organized meetings to harmonize cancer treatment guidelines—going beyond the previously developed NCCN Frameworks. The parent NCCN guidelines and the NCCN Frameworks provided an organized structure through which ACC panels could work in collaboration with NCCN experts to select their own SSA harmonized guidelines, beginning with the treatment of the 32 cancers with at least 1,000 incident cases in the WHO African (AFRO) region.^11^ As of August 2020, 46 NCCN Harmonized Guidelines for SSA have been posted on the NCCN website.^12^ This outcome represents a novel achievement in cancer management for SSA and provides a model for application in other resource-constrained regions of the world.

The collaborators who created the harmonized guidelines for SSA were motivated to provide tools for individual practitioners to prescribe evidence-based treatment strategies for patients in AFRO regions by addressing their unique needs and comorbidities. In this goal, they have succeeded. There are at least two additional benefits that go beyond this patient-specific application.

First, ministries of health and finance require accurate information about how much effective cancer care will cost and what value can be achieved with these health care investments. WHO has been working on modeling instruments to support priority setting, costing, and health system planning using a resource-stratified approach in line with World Health Assembly Resolution 70.12—tools that can provide meaningful data about health investment strategies.^13,14^ The NCCN Harmonized Guidelines either can provide defined patient pathways to structure economic costing models or can be used to validate the predictions of pre-existing models by assessing their relative accuracy.

Second, and of equal importance, the NCCN Harmonized Guidelines established resource-sensitive guidelines for the AFRO region that those SSA countries can embrace as their own evidence-based work product. Well-intending HIC partners can underappreciate the critical importance for country-specific projects to be driven by leadership from those countries. Paternalistic approaches to system design will not achieve the desired goal if the in-country partners do not embrace and accept the systematic approaches established through the partnerships. Though experts from other regions may have good knowledge about optimal cancer management in their own countries, they may be naïve to the systematic limitations that must be overcome in SSA countries. Only when the systematic approaches are adopted from within the SSA health care infrastructure can they be successfully sustained over time. At some point, the external partners are going to leave. If the cancer management approaches stop once those departures take place, then that is the definition of failure. Here, we have an example of an approach that is sustainable and truly African owned. This is the definition of success.
